# The effect of online collaborative writing instruction on enhancing writing performance, writing motivation, and writing self-efficacy of Chinese EFL learners

**DOI:** 10.3389/fpsyg.2023.1165221

**Published:** 2023-06-27

**Authors:** Yan Li

**Affiliations:** School of English Studies, Xi'an International Studies University, Xi'an, Shaanxi, China

**Keywords:** online collaborative writing instruction, writing performance, writing self-efficacy, writing motivation, EFL learners, Tencent docs

## Abstract

**Introduction:**

This study investigates the influence of online collaborative writing instruction on the writing performance, writing self-efficacy, and writing motivation of Chinese English as a foreign language (EFL) learners. The research was conducted at a language school in mainland China with 58 participants divided into an experimental group (n=30) and a control group (n=28).

**Methods:**

The experimental group utilized Tencent Docs, an online platform, for collaborative writing and peer-editing activities outside the classroom, while the control group received traditional in-class instruction. The study spanned a duration of 13 weeks, during which writing tasks, writing motivation scales, and writing self-efficacy scales were employed to collect data.

**Results:**

The findings revealed that the experimental group exhibited significantly greater improvement in writing performance, motivation, and self-efficacy compared to the control group. These results indicate the positive impact of incorporating Tencent Docs into collaborative writing instruction.

**Discussion:**

The outcomes of this study provide valuable insights for language educators regarding the benefits of integrating online tools into EFL instruction to enhance writing skills. By leveraging platforms like Tencent Docs for collaborative writing, instructors can foster improved performance, increased motivation, and enhanced self-efficacy among EFL learners. Overall, this research highlights the effectiveness of online collaborative writing instruction and its potential as a valuable tool for language educators seeking to optimize EFL learners’ writing abilities.

## Introduction

English as a Foreign Language (EFL) writing is a critical component of language proficiency and has received considerable attention from researchers in the field of second language (L2) education (Hwang et al., 2014). The increasing importance of English in the global arena has led to a growing interest in the teaching and learning of English writing (Zhao, 2010). Writing, being a complex and dynamic task, requires a significant amount of effort and practice to become proficient. In recent years, technology has revolutionized the way English is taught, and technology-assisted writing instruction has become a critical component of EFL writing (Barrot, 2021; Cancino and Panes, 2021; Loncar et al., 2021; Lin et al., 2022).

The increasing use of technology and the Internet has had a significant impact on language education, including EFL instruction (Hung, 2021). The use of online tools and platforms, such as wikis, blogs, podcasts, and Google Docs, has gained popularity as a means of creating interactive and collaborative writing environments for EFL students (Strobl, 2014; Dizon, 2016; Ebadi and Rahimi, 2017; Reinhardt, 2019; Saricaoglu, 2019; Xu et al., 2019; Hafner and Ho, 2020; Fathi et al., 2021; Hung et al., 2022). These tools allow students to practice English in an engaging and dynamic setting, while also promoting active learning, teamwork, and the development of social skills (Ravid et al., 2008; Xu et al., 2019; Barrot, 2021; Liu et al., 2023). The notion of using technology to support writing instruction via online collaborative writing is becoming increasingly appealing to EFL educators, as it offers solutions to limitations in time and space in traditional learning settings (Xu, 2021; Rahimi and Fathi, 2022).

The online environment provides opportunities for learners to receive immediate and constructive feedback from their peers and instructors, and engage in peer-editing and revision activities. This learning context also provides students with access to a range of resources, such as online dictionaries, grammar checkers, and writing samples, that can help students develop their writing skills (Reinhardt, 2019; Hafner and Ho, 2020). Google Docs is one such platform that is well-suited for facilitating peer feedback and collaboration among students (Ebadi and Rahimi, 2017; Fathi et al., 2021; Hoang and Hoang, 2022). The platform provides an easy means of uploading, sharing, and editing documents, allowing students to work together in real time and enhancing their learning experience (Yang, 2010; Liu and Lan, 2016; Ebadi and Rahimi, 2017). The use of such online collaborative writing platforms as a tool for writing instruction also offers benefits for EFL teachers, allowing them to encourage free expression and provide timely feedback (Chukharev-Hudilainen and Saricaoglu, 2016; Lai et al., 2016; Cho, 2017; Li, 2018; Fathi et al., 2021).

In this study, we investigate the impact of using a word processing tool on the writing performance of Chinese EFL learners. Specifically, we examine the effects of Tencent Docs, a commonly used word processing tool in China, on the writing development participants. It is worth mentioning that Tencent Docs, while having most of the features of Google Docs, was used in this study instead of Google Docs. This is because Google Docs is not widely used in China and Tencent Docs is the most commonly used alternative. Tencent Docs is a popular software suite used for productivity and document management in China. As a platform that offers multiple functionalities, including word processing, spreadsheets, and presentations, Tencent Docs is well-suited for language learning. In particular, the word processing component provides a rich environment for English writing learning, particularly for Chinese EFL learners (Tan et al., 2022). This is due to the software’s various tools that support EFL learners as they work on writing assignments and develop their writing skills. For instance, Tencent Docs Tencent Docs includes spell checkers, grammar checkers, and text-to-speech technology, which can help learners identify and correct errors in their writing (Zou et al., 2021), as well as improve their pronunciation and intonation. Moreover, the software offers a range of formatting options, including font size, style, and color, which can help learners develop their writing style and improve the overall readability of their texts.

Additionally, Tencent Docs enables learners to easily save, store, and share their documents in the cloud, allowing them to collaborate with peers and receive feedback from their teachers. This feature facilitates peer review and feedback, which can help learners refine their writing skills and gain confidence in their abilities. Furthermore, Tencent Docs offers templates and sample documents that can help learners develop their writing skills in various genres, such as academic essays, business letters, and personal narratives.

Concerning L2 writing, previous research has shown that collaborative writing instruction can enhance students’ writing performance, motivation, and self-efficacy (e.g., Rahimi and Fathi, 2022). Nevertheless, exploring the effectiveness of using online collaborative writing tools, such as Tencent Docs, in L2 writing instruction is still under-researched. Although some studies have investigated the use of Google Docs and other collaborative writing tools (e.g., Ebadi and Rahimi, 2017; Zou et al., 2021; Hoang and Hoang, 2022), to the best of our knowledge, no previous studies have explored the use of Tencent Docs in L2 writing instruction.

Tencent Docs has several unique features that may contribute to the effectiveness of online collaborative writing instruction. Firstly, Tencent Docs is a widely used online writing tool in China, making it readily accessible for language learners in mainland China. Secondly, Tencent Docs offers a wide range of writing resources, such as dictionaries, grammar checkers, and online writing communities, that can facilitate the collaborative writing process (Tan et al., 2022). Thirdly, Tencent Docs allows for real-time collaboration and peer feedback, which can enhance students’ writing skills and engagement in the writing process (Ma and Au, 2014; Li et al., 2020).

As such, this study aims to address this research gap and investigate the effectiveness of using Tencent Docs as an online collaborative writing tool in enhancing Chinese EFL learners’ writing performance, writing self-efficacy, and writing motivation. Through examining the unique contributions of Tencent Docs to the collaborative writing process, this study aims to provide valuable insights for language educators on the advantages of using online tools in improving EFL learners’ writing skills.

The results of this study are expected to contribute to the literature on EFL writing instruction and provide valuable insights into the use of Tencent Docs as a tool to enhance writing performance, motivation, and self-efficacy of Chinese EFL learners. The findings of this study have implications for language educators, curriculum designers, and policy makers, who are interested in incorporating technology-assisted writing instruction into the EFL writing curriculum. As such, this study also seeks to answer the following research questions:

1. What is the effect of Tencent Docs on the writing performance of Chinese EFL learners?
2. What is the effect of Tencent Docs on the writing motivation of Chinese EFL learners?
3. How does the use of Tencent Docs impact the writing self-efficacy of Chinese EFL learners?

## Literature review

### Online collaborative writing

Online collaborative writing is a promising field of research in second language writing that involves learners working together to co-create a single text using computer-mediated communication technologies. With advancements in technology devices, computer-mediated collaborative writing (CMCW) has become a popular field of research for L2 writing (Li, 2018; Liu et al., 2023). CMCW allows learners to enjoy ample chances for interaction, writing independence, and time–space convenience. Numerous studies have explored the impact of CMCW on various aspects of writing, such as patterns of interaction, writing outcomes, learners’ attitudes, types of learning tasks, and writing processes (e.g., Wang, 2015; Wu et al., 2015; Bikowski and Vithanage, 2016; Cho, 2017; Hafner and Ho, 2020; Barrot, 2021; Hoang and Hoang, 2022). These studies have demonstrated that CMCW can improve students’ writing complexity, accuracy, and fluency, as well as their overall writing performance (Reinhardt, 2019; Xu, 2021; Fathi and Rahimi, 2022; Jiang and Eslami, 2022).

In comparison to traditional face-to-face classes, online learning has been criticized for its inadequate social interaction and its limitations in overcoming the physical distance between students owing to its asynchronous and text-based nature (Lai et al., 2016; Akcaoglu and Lee, 2018). To address these challenges, methods such as synchronous online interactions, forums, and collaborative learning tasks have been proposed (Tu and Corry, 2003; Liu and Lan, 2016). Many studies have documented the beneficial influences of these activities on fostering social presence and individual communications in online learning settings. These remedial activities in educational contexts might consist of online peer-editing using Google Docs, Facebook group interactions, online collaborative assessments, blog-mediated instruction, and virtual exchanges (e.g., Pham and Usaha, 2016; Ebadi and Rahimi, 2017; Bugden et al., 2018; O’Dowd et al., 2020; Tan et al., 2022).

The integration of online educational devices in classrooms has been proven to be an effective means of promoting collaboration (Herrington et al., 2014). Web 2.0 offers various opportunities for collaboration through tools including wikis, blogs, social networks, online forums, virtual exchanges, and electronic portfolios (O’Dowd, 2020; Rahimi and Fathi, 2022). One popular application among these few tools is Google Docs, which is free and user-friendly, making it very appropriate for learners with few technology-related competencies. Google Docs is frequently employed for collaborative writing activities as far as peer-editing or peer-reviewing activities are concerned (Abrams, 2016, 2019; Rahimi and Fathi, 2022). Nevertheless, recent research indicates that there is room for improvement in terms of interactivity and enjoyment, as well as in increasing students’ satisfaction and intentions to use this learning method (Dinh and Nguyen, 2020).

The process of collaborative writing, as described by Storch (2013), involves multiple learners working together to create a shared text through the exchange of information, negotiation, and decision-making. The end result reflects collective learning, and collaboration is a crucial aspect at every stage of the writing process, including planning, drafting, and editing. Li and Zhu (2013) have noted that consistent collaboration can lead to language learning opportunities, while Wang (2015) suggests that it can also foster a sense of collaboration among learners and result in improved learning outcomes (Bikowski and Vithanage, 2016). As stated by Kessler and Bikowski (2010), the evolution of collaborative writing has been influenced by technological advancements, as they provide new opportunities for collaboration. Collaborative writing has been shown to play a significant role in language development from both a socio-cultural perspective (Vygotsky, 1978) and in the context of L2 acquisition theories (Kang and Lee, 2019; Ellis, 2021). Despite the perception of writing as a solitary activity, research has demonstrated that collaborative writing can have a positive impact on language learners’ writing processes, including increased accuracy, fluency, syntactic complexity, and overall writing performance (Jekiel, 2014; Ellis, 2021). Collaboration also helps to alleviate anxiety and low confidence levels, provides opportunities for learners to support each other, co-construct their second language knowledge, and receive immediate feedback (Brooks and Swain, 2009; Storch, 2011, 2013; Ellis, 2021).

However, online collaborative writing instruction is a relatively new approach to teaching writing that has gained popularity in recent years (Bikowski and Vithanage, 2016; Cho, 2017; Abrams, 2019). Compared to traditional face-to-face collaborative writing instruction, it offers several advantages. First, it enables learners to communicate and collaborate with their peers in real-time, regardless of their geographical location (Hsu, 2020). This feature of online instruction allows learners to work together and receive feedback from peers who may not be physically present in the same location, thus broadening the scope of the collaborative writing experience. Second, online collaborative writing instruction provides learners with access to a wide range of online writing resources (Lai et al., 2016; Abrams, 2019). These resources include online dictionaries, grammar checkers, and online writing communities, which can be used to support learners’ writing development. By using these resources, learners can enhance their writing skills and overcome common writing challenges more effectively (Li and Zhu, 2013; Hoang and Hoang, 2022).

According to Li (2018), the study of computer-mediated/online collaborative writing (CMCW) has been significantly influenced by sociocultural theory and social constructivism. In this context, collaborative writing is viewed as a social activity in which students interact and support one another’s learning and writing development. The sociocultural theory proposed by Vygotsky (1978) emphasizes the role of interaction in the learning process, particularly the use of language as a mediating tool and the concept of scaffolding. In collaboration, one partner (the expert) supports the other partner (the novice) in their learning, and this support takes place within the Zone of Proximal Development (Vygotsky, 1978). The partners can switch roles, with the novice becoming the expert and vice versa, during the course of the collaboration (Wells, 1999). Collaboration and language mediation (Lantolf and Thorne, 2006) enable the partners to communicate and work together effectively on tasks such as knowledge construction, problem-solving, and meaning-making (Rassaei, 2014; Hafner and Ho, 2020; Hung and Nguyen, 2022).

The use of CMCW in L2 classrooms is on the rise, with an increasing recognition of the benefits brought about by Web 2.0 technologies (Cho, 2017; Li, 2018). The positive impact of computer-mediated communication (CMC) on the design of collaborative writing tasks and the collaboration process, as well as learners’ writing quality and motivation, has been demonstrated in a number of studies (e.g., Armstrong and Retterer, 2008; Wang, 2015; Ebadi and Rahimi, 2017; Barrot, 2021). For example, Li and Zhu (2013) found that the use of wikis provided learners with greater flexibility in collaborative writing, while Wang (2015) reported improved learning outcomes in writing content, structure, and grammatical accuracy when using wikis for collaborative writing.

Despite the growing recognition of the benefits of CMCW in EFL contexts (Aydın and Yıldız, 2014; Wang, 2015; Xu et al., 2019; Fathi and Rahimi, 2022), challenges remain in its implementation, particularly in areas such as China, where technical difficulties and limited exposure to educational technology have been identified as barriers to its use (Rao and Lei, 2014; Lai et al., 2016; Paul and Liu, 2017; Barrot, 2021). Benson (2019) highlights the need to consider contextual factors and individual differences when incorporating CMCW tasks into the classroom to enhance their ecological validity.

Several studies have found that web-based collaborative writing has a positive impact on the writing skills and competencies of EFL learners. Aydın and Yıldız (2014) conducted a study on the effect of three types of collaborative writing tasks on intermediate level university students using wikis in EFL learning classrooms. The results indicated that wiki-based collaborative writing tasks enhanced the use of grammatical structures and were enjoyed by students. Another study by Bikowski and Vithanage (2016) investigated the impact of in-class web-based collaborative writing tasks on individual writing scores of L2 writers. The collaborative web-based writing group showed significantly greater gains in their individual writing scores and valued the collaborative writing tasks overall. Lai et al. (2016) explored the nature of collaboration and perceived learning in wiki-based collaborative writing among university EFL learners. The study revealed that collaboration patterns featuring high equality and mutuality were associated with positive attitudes and perceived learning. Lastly, Selcuk et al. (2021) aimed to analyze Turkish high school EFL learners’ self-reported accounts of their writing process in English with the support of group leaders in a web-based collaborative writing activity. The study found that group leaders facilitated planning tasks, provided corrective feedback, and emotional support such as praise and motivational phrases that increased self-confidence and motivation toward writing in English. Also, Teng (2021) aimed to explore the benefits of using interactive whiteboard technology for collaborative writing among English language learners. The study included 120 EFL students, and the results indicated that the use of interactive whiteboard technology with collaborative writing instruction led to greater improvement in students’ writing performance compared to traditional whiteboard-integrated collaborative writing and traditional collaborative writing instruction without whiteboard technology. The study also found that learners who received interactive whiteboard-integrated collaborative writing instruction exhibited higher levels of metacognitive activities and were more engaged in coregulation. This study highlights the potential of interactive whiteboard-integrated collaborative writing instruction in promoting writing instruction and suggests that it should be considered in language learning classrooms.

#### Online collaborative writing and L2 writing affective factors

A growing amount of research shows that good L2 writing is significantly impacted by affective and non-cognitive factors since L2 writing is a cognitively demanding activity (Piniel and Csizér, 2015; Han and Hiver, 2018; Fathi et al., 2019). Psychological factors that are unique to L2 writing can considerably improve L2 learners’ writing abilities by influencing their level of commitment to creating better-quality drafts (Piniel and Csizér, 2015). In light of this, the current study focused on three affective aspects of writing performance, writing motivation, and writing self-efficacy.

The primary tenets of the overall concept of motivation in L2 education serve as the foundation for L2 writing motivation, and as a crucial component of effective L2 learning, motivation is conceived as a dynamic process that is subject to constant change (Dörnyei, 2001; Waller and Papi, 2017). L2 motivation is a dynamic construct that is influenced by both internal and external elements that are related to the learner’s particular sociocultural and contextual background (Kozaki and Ross, 2011; Fathi et al., 2019). According to Dörnyei (2001), motivation determines why L2 learners choose a certain activity, how long they are willing to continue doing it, and how much work they put into it. Dörnyei (2001) further stated that motivation is a dynamic process subject to ongoing change and is a crucial component of effective L2 learning. The predominant definition of motivation nowadays emphasizes its dynamic and situational aspect. According to this viewpoint, an L2 learner is extremely likely to be impacted by many contextually dependent motives at once, and those motives may vary over time (Keblawi, 2009; Dornyei, 2019). Studies have shown that motivation is an important factor in language learning (Oxford and Shearin, 1994; Waninge et al., 2014). According to Williams and Burden (1997), L2 motivation is impacted by both internal elements that are particular to each learner and external ones that are pertinent to the learner’s sociocultural context. Additionally, learning motivation consists of an effort, a desire to learn, as well as positive attitudes about learning. Since writers connect with others, express themselves, and appeal to people, writing motivation is crucial (Chen, 2016). Seyyedrezaie et al. (2016) examined how the writing process in the Google Docs environment affected Iranian EFL students’ writing performance and concluded that Google Docs had a significant impact on improving students’ writing abilities and writing performance. Moreover, Liu and Lan (2016) carried out a study to investigate students’ perceptions, motivation and collaboration while using Google Docs. The findings of this study revealed that collaborators produced better writing, were more motivated to learn new things, and had a more optimistic outlook on the educational process. Mudawe (2018) examined the instructional potential of Google Docs as a collaborative tool to enhance EFL and ESL students’ writing in a Saudi environment. The results revealed that Google Docs increased students’ ability to communicate better and that Google Docs enhanced their text editing and revision in a motivating setting. According to Yang (2010), the easy use of Google Docs, which enables students to collaborate on writing assignments without being constrained by time or location, increases students’ motivation.

Self-efficacy is concerned with one’s perceptions of their own capacity to complete a certain learning activity. In a variety of academic situations, self-efficacy beliefs are thought to be extremely important for boosting students’ interest in learning (Bandura, 1997; Dornyei and Ryan, 2015). A great amount of empirical research within the L2 learning area indicates that strong self-efficacy is favorably connected with task performance and L2 skills (e.g., Pajares, 2003; Hsieh and Kang, 2010; Woodrow, 2011). Lower writing anxiety, a better sense of oneself as a writer, and a higher estimation of the importance of writing have all been linked to writing self-efficacy (Pajares, 2003; Fathi et al., 2019). According to a research by Piniel and Csizér (2015), writing self-efficacy is also positively connected with the learner’s interest and perseverance, self-regulatory ability, writing self-concept, goal achievement, and good writing performance. Also, according to Han and Hiver (2018), writing self-efficacy is defined as L2 learners’ views and confidence in their skills as L2 writers. According to social cognitive theory, self-efficacy is the belief and assessment that a person has regarding their capacity to perform at a given level and achieve particular objectives. Self-efficacious people establish tough objectives, are deeply dedicated to accomplishing them, are prepared to put up a considerable deal of effort to continue achieving particular objectives despite obstacles, and regain their feeling of effectiveness if they fail. According to Pajares (2003), mastery experiences have the greatest impact on how people perceive their level of self-efficacy because success increases it while failure decreases it. As a result, a person’s level of self-efficacy in writing is based on how confident they are in their ability to produce a particular kind of text. Moreover, Schunk and Zimmerman (2012) found that self-efficacious writers prefer to write more regularly and stick with writing assignments more frequently than students who have poor self-esteem. In other words, self-efficacy beliefs are substantially connected with students’ success and performance on writing activities. Students who feel self-sufficient in a writing course are more likely to choose writing projects and maintain an interest in completing them (Bandura, 1997; Tsiakyroudi, 2018). In addition, a self-efficacious writer shows strong enthusiasm for writing assignments, a pleasant attitude toward writing, and low writing anxiety (Fathi and Rahimi, 2022).

The literature on collaborative writing has highlighted the importance of peer interaction in improving writing performance among L2 learners (e.g., Li and Zhu, 2013; Bikowski and Vithanage, 2016). However, despite the numerous studies that have examined the impact of collaborative writing on L2 writing development (Storch, 2013), there remains a need for research that explores the effectiveness of different online collaborative writing tools in facilitating this process. Specifically, there is a dearth of studies that investigate the impact of using Tencent Docs for online collaborative writing instruction in L2 writing instruction. This study aims to fill this research gap by examining the impact of using Tencent Docs for online collaborative writing and peer-editing tasks on Chinese EFL learners’ writing performance, writing self-efficacy, and writing motivation. The purpose of this study is to investigate whether the use of Tencent Docs as a collaborative writing tool can result in greater improvements in writing performance, writing self-efficacy, and writing motivation than traditional face-to-face collaborative writing instruction.

## Methods

### Participants

A total of 58 intermediate Chinese EFL learners, selected via convenience sampling method, participated in this study. The participants were students of two intact classes and were assigned to two groups, with 30 participants in the experimental group and 28 participants in the control group. These participants were a mix of male and female students, and the age range was between 18 and 22 years. The study was conducted at a language school located in in mainland China. All participants had prior learning experience in English, with their native language being Mandarin Chinese. In order to ensure homogeneity among the participants, a DIALANG test^1^ was administered to them. Only those participants whose proficiency level, as assessed by the DIALANG test, was B1 (intermediate) were included in the study.

### Instruments

#### Writing performance test

A standardized writing test was administered to assess the writing performance of the participants. The test consisted of a prompt and a writing task that was designed to measure the participants’ ability to generate ideas, organize their thoughts, and produce a well-written piece of text. The pre- and post-test writing assessments each consisted of a writing prompt, which required participants to write argumentative essays. The writing prompts and the duration of writing (40 min) were selected from the Independent Writing section of the TOEFL internet-based test. Both prompts were related to foreign language learning, aligning with the theme of the Tencent Docs-based collaborative writing tasks. The questions aimed to evaluate participants’ ability to express their opinions and support them with specific reasons and evidence, which would reflect any improvements made through writing argumentative essays in the Tencent Docs-based collaborative writing tasks. It is worth noting that these tasks were independent of the four treatment writing tasks which were completed during the writing course.

**Pre-test prompt:** Do you think it is necessary for children to learn foreign languages in primary school? Why or why not? Use specific reasons and evidence to support your answer.

**Post-test prompt:** Some people believe that the best way to learn a foreign language is to immerse oneself in a country where the language is spoken, while others think that learning through technology is just as effective. What is your opinion and why? Use specific reasons and evidence to support your preferred choice.

#### Writing self-efficacy scale

In this study, the Writing Self-Efficacy Scale (see Appendix A) constructed by Han and Hiver (2018) was applied to assess the writing self-efficacy of EFL students. The scale, consisting of seven items adapted from Mills et al. (2006), aimed to evaluate the confidence and beliefs of the L2 students in their writing skills. The scale was designed in the form of a 5-point Likert questionnaire, where 1 indicated “strongly disagree” and 5 indicated “strongly agree.” The reliability of the scale was determined using Cronbach’s Alpha and was reported to be 0.83.

#### Writing motivation scale

The scale used in the study was the L2 Writing Motivation Scale (see Appendix B) developed by Waller and Papi (2017). This scale includes seven items related to L2 writing motivation, developed based on other general L2 motivation scales created by Taguchi et al. (2009). The scale encompasses statements related to the motivation of L2 writers in learning the language, their desire, and their motivation intensity. Each item was rated on a 6-point Likert scale, ranging from 1 (never) to 6 (always). The reliability of the scale, calculated using Cronbach’s Alpha, was 0.81 in the present research.

#### Application

The experimental group used Tencent Docs for online collaborative writing and peer-editing tasks outside of the classroom. Tencent Docs is a free, multi-functional office suite developed by Tencent, a Chinese multinational conglomerate. This application provides features such as document collaboration, real-time co-authoring, and revision history, which were ideal for the needs of the experimental group. The control group did not use any online writing tool, and their writing instruction was limited to in-class activities and homework assignments.

### Procedure

The writing program, which was conducted over a duration of 13 weeks, involved both the experimental and control groups receiving in-class instruction. The course was structured according to the principles of the process approach and was taught by a single educator, who employed the same materials and curriculum for both groups. The primary objective of the course was to introduce the students to various forms of paragraphs, including descriptive and process, opinion, comparison/contrast, and solution paragraphs.

The students in the experimental group received in-class instruction and were required to use Tencent Docs for online collaborative writing and peer-editing of their written tasks outside the classroom. The writing process started in the classroom, where each student wrote the first draft of their written task. The peer-editing process took place outside the classroom, where students shared their work on Tencent Docs and received feedback and comments from their peers. The students were divided into groups of four or five and were instructed to share comments and peer-edit each other’s written work on Tencent Docs. Each group collaborated on one single writing assignment. The students wrote their first draft individually and shared it with their peers on Tencent Docs. They then provided feedback and made suggestions on each other’s writing by leaving comments on the document, and also used the “Track Changes” feature to directly edit the document. After receiving feedback from their peers, the students revised their writing and produced a second draft, which they shared with their peers for further feedback. This process continued until the final draft was written. In this process, they received peer feedback and edited each other’s writing based on the components of content, organization, language use, vocabulary, and mechanics.

The students in the control group were also divided into groups of four or five and were required to engage in peer-editing in a collaborative way. However, they did not use any technology devices such as Tencent Docs. Instead, they performed the peer-editing process by physically exchanging their written work and providing each other with feedback and comments. In both groups, the participants were first introduced to the process of peer-editing and collaborative writing. The educator furnished the students with an exemplar video in which a professional evaluator demonstrated the complete procedure of peer-reviewing a written document. The students were also given comprehensive explanations of the aspects of writing by the educator. The students in both groups went through several drafts of their written work, starting with the first draft and receiving feedback, before finally producing the final draft. The teacher and peers provided feedback on each draft until the final draft was written.

To ensure consistency in the peer-editing process, both the experimental and control groups received training on how to give feedback in class. The teacher provided guidance on how to identify areas for improvement in their peers’ writing and how to provide constructive feedback. The students were also given a rubric that outlined the different aspects of writing, such as content, organization, language use, vocabulary, and mechanics, to guide them in their peer-editing.

In terms of the frequency and number of feedback provided for each group, each student in the experimental group received feedback from three to four peers, while each student in the control group received feedback from four to five peers. The number of feedback provided was not significantly different between the two groups. In total, the students in both groups completed four writing tasks during the 13-week writing program. For each writing task, the students were given a minimum of 2 weeks to complete, and the students in both groups spent approximately the same amount of time on each task.

### Data analysis

To assess the effect of online collaborative writing instruction on writing performance, writing motivation, and writing self-efficacy, three ANCOVAs were conducted. The first ANCOVA was conducted to compare the writing performance scores between the experimental group and the control group. The second ANCOVA was conducted to compare the writing motivation scores between the two groups. The third ANCOVA was conducted to compare the writing self-efficacy scores between the two groups. The results of the ANCOVAs were reported using means, standard deviations, and effect sizes, and the level of significance was set at *p* < 0.05.

Also, the writing scoring rubric developed by Jacobs et al. (1981) was used to assess the writing performance of the participants in both the pre-test and post-test. This analytical scoring technique considers various criteria, including content, organization, vocabulary use, language use, and mechanics, in order to score an essay. To validate the scoring process, a trained rater was assigned to score a third of the written tasks, and the results were compared with those of the primary rater. Both raters were blind to the experimental treatment to prevent any potential bias in the scoring process. More precisely, neither the primary rater nor the trained rater knew which group the participants belonged to, whether they were in the experimental or control group. This blinding process aimed to reduce the potential for systematic errors or biases in the scoring process. The Cohen’s Kappa inter-rater reliability test indicated a high degree of consistency in the scoring process, with a reliability index of 0.84.

## Results

First, descriptive statistics were calculated for the participants’ scores on pre- and post-tests of the dependent variables (as seen in Table 1). Then the researcher used ANCOVA to compare the effects of two types of EFL writing instruction (online collaborative course and traditional) on the writing performance, as well as its sub-scales, writing motivation, and writing self-efficacy of the EFL students. ANCOVA is appropriate for this research because it allows the researcher to control for any pre-existing differences between the groups by using the scores on the pre-tests as a covariate. By doing so, the researcher can determine if any observed differences in writing performance, writing motivation, and writing self-efficacy can be attributed to the type of instruction received and not to pre-existing differences between the groups.

**Table 1:** Descriptive statistics of writing skills, holistic writing, writing motivation, and writing self-efficacy for both groups.

	Group	*N*	Mean	Std. deviation	Std. error mean
Content1	Experimental	30	13.0050	2.31466	0.42260
	Control	28	12.7500	1.96968	0.37223
Content2	Experimental	30	17.1998	3.34222	0.61020
	Control	28	14.1736	2.57358	0.48636
Organization1	Experimental	30	11.5259	1.52394	0.27823
	Control	28	11.2054	1.49574	0.28267
Organization2	Experimental	30	15.6060	2.77759	0.50712
	Control	28	13.5750	2.80339	0.52979
Language1	Experimental	30	13.2548	1.75253	0.31997
	Control	28	12.8862	1.72010	0.32507
Language2	Experimental	30	16.8192	2.33682	0.42664
	Control	28	14.5487	1.98730	0.37556
Vocabulary1	Experimental	30	7.9817	2.22261	0.40579
	Control	28	7.4118	2.54629	0.48120
Vocabulary2	Experimental	30	10.1829	2.71970	0.49655
	Control	28	8.3430	2.69091	0.50853
Mechanics1	Experimental	30	5.2867	1.40975	0.25738
	Control	28	5.6596	1.50777	0.28494
Mechanics2	Experimental	30	7.0539	1.78201	0.32535
	Control	28	5.9907	1.89296	0.35774
Writing^1^1	Experimental	30	54.4833	9.32967	1.70336
	Control	28	56.2143	12.18790	2.30330
Writing2	Experimental	30	69.9000	10.20260	1.86273
	Control	28	63.9286	12.16226	2.29845
Motivation1	Experimental	30	18.4667	4.41770	0.80656
	Control	28	19.7500	2.82023	0.53297
Motivation2	Experimental	30	24.2833	4.26631	0.77892
	Control	28	21.3929	2.76338	0.52223
Self.efficacy1	Experimental	30	19.9833	4.56464	0.83339
	Control	28	20.8571	3.24852	0.61391
Self.efficacy2	Experimental	30	28.8833	7.72555	1.41049
	Control	28	23.0893	3.90644	0.73825

1 It refers to holistic writing.

Table 1 shows the descriptive statistics of writing subscales, holistic writing, writing motivation, and writing self-efficacy for both experimental and control groups. For content, the pre-test mean score for experimental group (M = 13.01, SD = 2.31) was slightly higher than the control group (M = 12.75, SD = 1.97). However, for content post-test, the mean score for the experimental group (M = 17.19, SD = 3.34) was higher than the control group (M = 14.17, SD = 2.57). In terms of organization, the mean score for experimental group on the pre-test (M = 11.52, SD = 1.52) was slightly higher than the control group (M = 11.20, SD = 1.49). Nevertheless, for organization post-test, the mean score for the experimental group (M = 15.60, SD = 2.77) was substantially higher than the control group (M = 13.57, SD = 2.80). Concerning language use, the pre-test mean score for experimental group (M = 13.25, SD = 1.75) was slightly higher than the control group (M = 12.88, SD = 1.72). However, for language post-test, the mean score for the experimental group (M = 16.81, SD = 2.33) was significantly greater than the control group (M = 14.54, SD = 1.98).

Regarding vocabulary, the mean score for experimental group on the pre-test (M = 7.98, SD = 2.22) was not much higher than the control group 1 (M = 7.41, SD = 2.54). For vocabulary post-test, the mean score for the experimental group (M = 10.18, SD = 2.71) was significantly higher than the control group (M = 8.34, SD = 2.69). For mechanics, the mean score for experimental group on the pre-test (M = 5.28, SD = 1.40) was similar to the control group 1 (M = 5.65, SD = 1.50). Nonetheless, for mechanics post-test, the mean score for the experimental group (M = 7.05, SD = 1.78) was greater than the control group (M = 5.99, SD = 1.89). As for holistic writing, the mean score for the experimental group on the post-test (M = 69.90, SD = 10.20) was significantly higher than the control group (M = 63.92, SD = 12.16).

Also, the mean scores for writing motivation pre-test were 18.46 (SD = 4.41) and 19.75 (SD = 2.82) for the experimental and control groups, respectively. For writing motivation post-test, the mean scores were 24.28 (SD = 4.26) for the experimental group and 21.39 (SD = 2.76) for the control group. The mean scores for writing self-efficacy pre-test were 19.98 (SD = 4.56) and 20.85 (SD = 3.24) for the experimental and control groups, respectively. Finally, the mean scores for writing self-efficacy post-test were 28.88 (SD = 7.72) for the experimental group and 23.08 (SD = 3.90) for the control group.

In ANCOVA analysis, the results were adjusted for pre-existing differences between the groups by considering the scores on the pre-test as a covariate. The independent variable in the analysis was the type of intervention (online collaborative or traditional), while the dependent variable consisted of scores on holistic writing performance, as well as its sub-tests, motivation, and self-efficacy obtained after the completion of the treatment. Prior to conducting the ANCOVAs, various validity tests were performed to verify that the data met the conditions of normal distribution, linearity, equal variances, equal regression slopes, and accurate measurement of the covariate.

The results of the analysis of the effect of the online collaborative writing course on students’ L2 writing performance showed that the writing performance of the experimental group improved significantly more than that of the control group. The writing performance mean score of the experimental group increased from 54.48 on the pre-test to 69.90 on the post-test, while the pre-test mean score of the control group increased from 56.21 to 63.92 on the post-test. However, after controlling for pre-test scores (see Table 2), the difference between the two groups was found to be statistically significant [*F*(1, 55) = 10.44, *p* = 0.002, partial eta squared = 0.16]. This suggests that the online collaborative writing course was effective in enhancing the students’ holistic writing performance.

**Table 2:** The results of ANCOVA on holistic writing performance.

Source	Type III sum of squares	Df	Mean square	F	Sig.	Partial eta squared
Pre writing	3119.936	1	3119.936	44.082	0.000	0.445
Group	739.104	1	739.104	10.443	0.002	0.160
Error	3892.621	55	70.775			

Additionally, to examine the effect of the online collaborative writing on the writing sub-scales, a series of ANCOVAs were performed. The results in Table 3 show that there is a significant effect of group on the content component of writing, *F*(1, 55) = 46.521, *p* < 0.001, partial eta squared = 0.458, providing evidence that online collaborative writing instruction has a positive effect on enhancing the content component of writing, and this effect is statistically significant even after controlling for the pretest score.

**Table 3:** Results of ANCOVA for writing content.

Source	Type III sum of squares	Df	Mean square	F	Sig.	Partial eta squared
Content1	376.602	1	376.602	164.168	0.000	0.749
Group	106.718	1	106.718	46.521	0.000	0.458
Error	126.170	55	2.294			

The results of ANCOVA for writing organization are presented in Table 4. The main effect of Group was also significant, *F*(1, 55) = 9.086, *p* = 0.004, partial eta squared = 0.458 = 0.142, indicating that there was a significant difference between the experimental and control groups in writing organization, with the experimental group outperforming the control group.

**Table 4:** Results of ANCOVA for writing organization.

Source	Type III sum of squares	Df	Mean square	F	Sig.	Partial eta squared
Organization1	207.926	1	207.926	50.157	0.000	0.477
Group	37.666	1	37.666	9.086	0.004	0.142
Error	228.002	55	4.145			

Table 5 shows the results of the ANCOVA to examine the effect of online collaborative writing instruction on writing language use, with language use pre-test scores as a covariate. The main effect of group was also significant, *F*(1, 55) = 65.549, *p* < 0.001, partial η^2^ = 0.544, suggesting that the experimental group outperformed the control group in writing language use. The effect size was moderate. The interaction effect between language use pre-test scores and group was not significant, *F*(1, 55) = 0.072, *p* = 0.790, partial η^2^ = 0.001, indicating that the effect of the online collaborative writing instruction on writing language use was not influenced by the initial language use ability of the participants.

**Table 5:** Results of ANCOVA for writing language use.

Source	Type III sum of squares	Df	Mean Square	F	Sig.	Partial eta squared
Language1	224.060	1	224.060	301.055	0.000	0.846
Group	48.785	1	48.785	65.549	0.000	0.544
Error	40.934	55	0.744			

Based on the results presented in Tables 6, a significant main effect of vocabulary was observed, *F*(1, 55) = 762.696, *p* < 0.001, partial η^2^ = 0.933. Additionally, a significant main effect of group was observed, *F*(1, 55) = 42.035, *p* < 0.001, partial η^2^ = 0.433. The Type III Sum of Squares for vocabulary1 was 382.436, indicating that the variation in writing vocabulary scores could be attributed to the independent variable. The Type III Sum of Squares for group was 21.078, indicating that the group factor accounted for some of the variance in writing vocabulary scores. The error term was 27.578.

**Table 6:** Results of ANCOVA for writing vocabulary.

Source	Type III sum of squares	df	Mean square	F	Sig.	Partial eta squared
Vocabulary1	382.436	1	382.436	762.696	0.000	0.933
Group	21.078	1	21.078	42.035	0.000	0.433
Error	27.578	55	0.501			

A one-way ANCOVA was performed to determine the effect of the writing intervention program on writing mechanics. The results are presented in Table 7. The main effect of Mechanics1 was statistically significant, *F*(1, 55) = 496.886, *p* < 0.001, with a large effect size, partial eta squared = 0.900. This indicates that the writing intervention program had a significant effect on the participants’ writing mechanics scores. Additionally, the main effect of Group was also statistically significant, *F*(1, 55) = 94.776, *p* < 0.001, with a moderate effect size, partial eta squared = 0.633, suggesting that there were significant differences in writing mechanics scores between the two groups. The interaction between Mechanics1 and Group was not statistically significant, *F*(1, 55) = 1.426, *p* = 0.237, indicating that the effect of the writing intervention program did not differ significantly between the two groups in terms of writing mechanics.

**Table 7:** Results of ANCOVA for writing mechanics.

Source	Type III sum of squares	df	Mean Square	F	Sig.	Partial eta squared
Mechanics1	170.021	1	170.021	496.886	0.000	0.900
Group	32.430	1	32.430	94.776	0.000	0.633
Error	18.820	55	0.342			

Based on the data analysis, which included descriptive statistics and ANCOVA, the results of this study suggest that the online collaborative writing instruction significantly contributed to enhancing the holistic writing and its sub-scales including content, organization, language use, vocabulary, and mechanics. Therefore, it can be concluded that the intervention had a positive impact on various writing components and can be considered as an effective approach to enhance students’ writing performance.

Concerning the effect of online collaborative writing instruction on EFL learners’ L2 writing motivation, the descriptive statistics (Table 1) revealed that the pre-test mean score for the experimental group was 18.46 and increased to 24.28 on the post-test, while the pre-test mean score for the control group was 19.75 and increased to 21.39 on the post-test. However, after taking into consideration the pre-test scores of writing motivation, a significant difference was found between the two groups on post-test scores, as shown in Table 8 [*F*(1, 55) = 12.88, *p* = 0.001, partial eta squared = 0.19]. This result implies that the experimental group’s writing motivation improved more significantly than the control groups, implying the effectiveness of the online collaborative writing course in promoting writing motivation among students.

**Table 8:** The results of ANCOVA on writing motivation.

Source	Type III sum of squares	df	Mean square	F	Sig.	Partial eta squared
Pre motivation	80.236	1	80.236	6.750	0.012	0.109
Group	153.217	1	153.217	12.889	0.001	0.190
Error	653.784	55	11.887			

Finally, according to the descriptive statistics in Table 1, the writing self-efficacy score for the control group increased from a pre-test mean score of 20.58 to a post-test mean score of 23.08. Meanwhile, the writing self-efficacy score for the experimental group rose from a pre-test mean of 19.98 to a post-test mean of 28.88. Further analysis through ANCOVA, taking into consideration the pre-test scores of writing self-efficacy, showed a statistically significant difference in the post-test scores of writing self-efficacy between the two groups, with *F*(1, 55) = 17.36, *p* = 0.000, and partial eta squared = 0.24 (see Table 9). This result implies that the online collaborative writing course had a positive impact on the writing self-efficacy of the students.

**Table 9:** The results of ANCOVA for writing self-efficacy.

Source	Type III sum of squares	df	Mean square	F	Sig.	Partial eta squared
Pre self-efficacy	329.830	1	329.830	10.006	0.003	0.154
Group	572.499	1	572.499	17.367	0.000	0.240
Error	1813.038	55	32.964			

## Discussion

The present study sought to examine the effect of online collaborative writing using Tencent Docs on the writing performance, writing motivation, and writing of Chinese EFL learners. The results of the data analysis for this quasi-experimental design indicated that online collaborative writing instruction improved L2 writing performance of EFL learners. This outcome is in line with empirical research evidence reported in the literature (e.g., Aydın and Yıldız, 2014; Bikowski and Vithanage, 2016; Lai et al., 2016; Li, 2018; Kang and Lee, 2019; Hsu, 2020; Selcuk et al., 2021; Teng, 2021; Fathi and Rahimi, 2022; Jiang and Eslami, 2022; Rahimi and Fathi, 2022), which highlighted the significant role of online collaborative writing in enhancing writing outcomes. This finding lends more credit to the idea that the use of online tools, such as Tencent Docs, can play a valuable role in enhancing writing skills among EFL learners (Cancino and Panes, 2021). The technology-enhanced writing environment may provide opportunities for real-time collaboration, revision, and feedback, which can positively impact writing performance (Pham, 2020; Barrot, 2021). Additionally, the use of Tencent Docs may increase the accessibility and frequency of writing activities, leading to more writing opportunities and experiences (Lin et al., 2022). Tencent Office offers the possibility of giving and receiving peer evaluation, instead of writing solely for teachers, students were writing for a large audience, and this may lead them to keep their efforts to produce better drafts feedback (Pham and Usaha, 2016; Cho, 2017; Ebadi and Rahimi, 2017). Additionally, the use of Tencent Docs may increase the accessibility and frequency of writing activities, leading to more writing opportunities and experiences (Lin et al., 2022). Tencent Office offers the possibility of peer evaluation, allowing students to receive feedback from a large audience instead of writing solely for their teachers. This may motivate students to put more effort into producing better drafts and seeking feedback to improve their writing skills (Pham and Usaha, 2016; Cho, 2017; Ebadi and Rahimi, 2017). However, it is important to note that while peer evaluation can enhance students’ feedback practices, it is the writing instruction and the teacher’s guidance that can ultimately lead to improved writing performance (Hedgcock and Lefkowitz, 1996). Therefore, incorporating Tencent Docs into writing instruction should be accompanied by effective teaching strategies and teacher support to maximize its potential benefits for students’ writing development.

Furthermore, the use of Tencent Docs may enhance collaborative learning and increase the accessibility and frequency of writing activities, providing students with more opportunities to practice their writing skills (Lin et al., 2022). Tencent Office also facilitates peer evaluation, which allows students to receive feedback from their peers and write for a larger audience (Pham and Usaha, 2016; Cho, 2017; Ebadi and Rahimi, 2017). This process encourages students to engage in more critical thinking and self-reflection, leading to the production of higher quality writing samples (Cho, 2017; Ebadi and Rahimi, 2017). Therefore, the use of Tencent Docs can promote both writing performance and feedback practice, providing a comprehensive approach to writing instruction.

As a result, a more engaging and interactive writing place can be found in the Tencent Office environment and individuals were more inspired to publish higher-quality writing drafts. In addition, as this online collaborative learning environment removes the time and place limitation (Yang, 2010; Wang, 2015; Xu et al., 2019), students may have more opportunities for writing practice, brainstorming, reviewing, and revising their writing beyond the class walls. This positive effect of online collaborative writing has been acknowledged in the literature (e.g., Bikowski and Vithanage, 2016; Reinhardt, 2019; Barrot, 2021; Su and Zou, 2022). Moreover, the students’ overall positive attitudes toward using online collaborative learning platforms in L2 learning contexts might have enhanced their writing performance. Likewise, Zhou et al. (2012) revealed that students had positive attitudes toward communicating with numerous peers on Google Docs, therefore, they focused more on the quality of their written assignments and as a result, their writing performance improved. In partial agreement with Seyyedrezaie et al. (2016), the results showed that the writing process in the Google Docs environment significantly affected students’ writing abilities and writing performance. Overall, the use of Tencent Docs provides a number of features and tools that can support and facilitate the writing process, including text-to-speech functions, spell checking, and thesaurus tools, which can help learners to improve their writing accuracy and fluency.

Second, the results revealed that online collaborative writing instruction using Tencent Docs improved L2 writing motivation. This result is in accordance with the findings of some previous studies (e.g., Yang, 2010; Liu and Lan, 2016; Mudawe, 2018; Yousefifard and Fathi, 2021). Since writers communicate, express themselves, and appeal to people while using Tencent, writing motivation is crucial and students with writing motivation had high levels of effort, desire to write, as well as positive attitudes about writing. In line with what Godwin-Jones (2018) concluded, it was revealed that students’ motivation and higher-order thinking abilities were increased through online collaborative peer editing. Using Tencent, students were willing to continue writing, and invest a large amount of time, resulting in higher writing motivation. Also, in harmony with what Yang (2010) claimed, as a result of the using online collaborative writing, which helps students to work on writing assignments without being limited by time or location, increases students’ motivation. In addition, Tencent provided a motivating environment (Mudawe, 2018; Luo et al., 2022) for students to freely edit and revise their drafts, communicate and share ideas. Generally, it was revealed that with the use of online collaborative writing, students had a more positive attitude toward writing and experienced higher levels of motivation to write (Liu and Lan, 2016). This finding also aligns with previous research, which has demonstrated that the use of technology in the language learning process can increase learners’ motivation and engagement (Sun and Gao, 2020; Su and Zou, 2022). The use of Tencent Docs provided a dynamic and interactive environment (Ng et al., 2022) that can enhance learners’ writing motivation by making the writing process more enjoyable and interesting. In other words, the online environment may provide a more engaging and interactive writing experience, as well as opportunities for receiving feedback and recognition from peers and instructors, which can boost writing motivation.

Third, the findings indicated that online collaborative writing instruction improved L2 writing self-efficacy of the participants. This finding supports the idea that the use of online collaborative writing tools, such as Tencent Docs, can enhance writing self-efficacy beliefs among EFL learners. In line with Rahimi and Fathi (2022), it can be argued that the online collaborative learning environment may provide opportunities for real-time collaboration, revision, and feedback, which can increase the confidence and competence of EFL learners in writing. Following Lee and Evans (2019), it can be argued that the further feedback and comments on tasks provided by Tencent Office might have enhanced writing self-efficacy of the participants. It is important to note that the software allows for real-time collaborative writing and editing, as well as the option for peer review and feedback. With the peer review feature, students are able to provide feedback and suggestions to each other, which can enhance their sense of self-efficacy as they feel more in control of their learning and confident in their ability to produce quality work. Additionally, the software provides a variety of tools and resources, such as templates and formatting options, which can assist students in the writing process and boost their confidence in their ability to complete writing tasks. These features of Tencent Docs may have contributed to the enhancement of writing self-efficacy among the participants. Additionally, the online environment may provide access to a wider range of writing resources and materials, which can further enhance writing self-efficacy beliefs. This finding is also in harmony with the theoretical notion of self-efficacy construct (e.g., Schunk and Zimmerman, 2012; Piniel and Csizér, 2015).

Following Lee and Evans (2019), it can be argued that the further feedback and comments on tasks provided by Tencent Office might have enhanced writing self-efficacy of the participants. Tencent Docs offers a range of collaborative features, including the ability to share documents, comment on others’ writing, and receive feedback from peers and instructors. The software also provides a built-in spell checker, grammar checker, and word count tool, which may help students improve the accuracy and fluency of their writing. In addition, the online environment provides access to a wider range of writing resources and materials, such as online dictionaries, thesauruses, and writing guides, which can further enhance writing self-efficacy beliefs. This finding is also in harmony with the theoretical notion of self-efficacy construct (e.g., Schunk and Zimmerman, 2012; Piniel and Csizér, 2015).

To clarify the feedback and comments provided by Tencent Docs, it is important to note that the software allows for real-time collaborative writing and editing, as well as the option for peer review and feedback. With the peer review feature, students are able to provide feedback and suggestions to each other, which can enhance their sense of self-efficacy as they feel more in control of their learning and confident in their ability to produce quality work. Additionally, the software provides a variety of tools and resources, such as templates and formatting options, which can assist students in the writing process and boost their confidence in their ability to complete writing tasks. These features of Tencent Docs may have contributed to the enhancement of writing self-efficacy among the participants.

As a result of having strong self-efficacy beliefs, students’ interest in writing was substantially boosted. Self-efficacious students had pleasant attitudes toward writing assignments, low writing anxiety (Fathi and Rahimi, 2022), and a high level of enthusiasm for doing writing tasks. It can be argued that students who have high self-efficacy beliefs are more likely to set challenging goals, demonstrate a strong commitment to achieving them, and persevere in the face of obstacles. Furthermore, they may be better equipped to bounce back from setbacks and failures, as they are more likely to maintain their confidence and motivation. Such findings are consistent with the notion that self-efficacious students tend to approach academic tasks with a positive attitude and a willingness to invest effort in their learning (Piniel and Csizér, 2015).

These findings are aligned with the conclusion drawn by Pajares (2003) that the writing self-efficacy beliefs of L2 learners can have an impact on all other dimensions of their learning performance. Therefore, it can be concluded that a significant interplay can be found between self-efficacy beliefs and students’ performance on writing activities, because self-efficacious writers showed a positive attitude toward writing assignments, as well as a low level of anxiety when writing, resulting in enhanced writing performance. With the use of online collaborative writing, students had a better sense of themselves and their writings, and felt less anxious (Abrams, 2016, 2019; Fathi et al., 2019), which resulted in improved self-efficacy. Besides, as online collaborative writing improved students’ writing performance significantly, they felt more interested and motivated as a result of getting positive achievements. In other words, as students were motivated enough to put efforts into their writing process, their writing ability and creativity boosted significantly (Zhang et al., 2014; Yu et al., 2020). From a broader perspective, this finding supports previous research, which has indicated that the use of technology in the writing process can increase learners’ self-efficacy and confidence in writing (Lai et al., 2016; Li et al., 2020; Jiang and Eslami, 2022; Rahimi and Fathi, 2022). In fact, the use of Tencent Docs provided a supportive and user-friendly environment that can help learners to overcome writing challenges and feel more confident in their writing abilities. Overall, the results of the present study indicated the potential benefits of using Tencent Docs in the writing instruction of Chinese EFL learners. It was found that the use of this technology can positively impact writing performance, writing self-efficacy, and writing motivation.

## Conclusion

This study tried to investigate the effect of online collaborative writing using Tencent Docs on the writing performance, writing motivation, and writing self-efficacy of Chinese EFL learners. Having utilized a pre-test and post-test design with an experimental group and a control group, the researcher found that the use of Tencent Docs significantly improved the writing performance, writing self-efficacy, and writing motivation of the participants in the experimental group. These findings can contribute to the development of new teaching practices and technology-enhanced language learning programs, and can help to improve the writing skills of Chinese EFL learners. The results of this study support the idea that technology-enhanced writing instruction can have a positive impact on the writing skills of EFL learners. The use of Tencent Docs helped the participants to produce higher quality writing samples, which suggests that this software can provide valuable resources for improving writing skills. Furthermore, the positive impact on writing self-efficacy verifies the potential of technology-enhanced writing instruction to promote learner autonomy and self-directed learning. The findings might also have important implications for language teachers and educational institutions. By incorporating Tencent Docs into writing instruction, language teachers can provide students with a user-friendly and engaging tool that can enhance their writing performance, self-efficacy, and motivation. Furthermore, the results of this study suggest that the use of Tencent Docs can be particularly beneficial for Chinese EFL learners, who often struggle with writing skills in English.

The findings of this study have significant implications for the broader area of L2 writing literature and technology-enhanced writing instruction. By demonstrating the positive effects of online collaborative writing using Tencent Docs on writing performance, writing self-efficacy, and writing motivation, this study contributes to the growing body of research that advocates for the use of technology in language learning. The use of technology in writing instruction can enhance learners’ engagement, motivation, and autonomy by providing a platform for collaborative learning and feedback, as well as by offering a range of tools and resources to support writing skills development. The outcomes of this research also shed light on the significance of teacher training in technology-enhanced writing instruction, as teachers need to be equipped with the necessary skills and knowledge to effectively integrate technology into their teaching practices. Furthermore, the study highlights the importance of developing learner autonomy and self-directed learning in language education. Via employing technology such as Tencent Docs, EFL learners can engage in more independent and interactive writing activities, which can foster their self-efficacy and motivation for writing.

However, the present study might have some limitations that should be taken into consideration. Firstly, the sample size was relatively small, and future researcher could benefit from a larger sample size to increase the generalizability of the findings. Secondly, the study was conducted over a short time frame, and the impact of Tencent Docs on writing skills over a longer period of time should be explored in future studies. Finally, the study only examined the use of Tencent Docs in an English writing context, and future research could benefit from exploring its impact in other writing tasks, such as composing in Chinese.

## Data availability statement

The raw data supporting the conclusions of this article will be made available by the author, without undue reservation. Requests to access these datasets should be directed to YL, yanlee200909@163.com.

## Ethics statement

The studies involving human participants were reviewed and approved by School of English Studies, Xi’an International Studies University. The patients/participants provided their written informed consent to participate in this study.

## Author contributions

The author confirms being the sole contributor of this work and has approved it for publication.

## Conflict of interest

The author declares that the research was conducted in the absence of any commercial or financial relationships that could be construed as a potential conflict of interest.

## Publisher’s note

All claims expressed in this article are solely those of the authors and do not necessarily represent those of their affiliated organizations, or those of the publisher, the editors and the reviewers. Any product that may be evaluated in this article, or claim that may be made by its manufacturer, is not guaranteed or endorsed by the publisher.

## Figures and Tables

**Table:** 

Items	Strongly disagree	Disagree	Neutral	Agree	Strongly agree
1	I feel confident about writing in English.				
2	I know how to write well in English.				
3	I write in English with an underlying logical organization.				
4	If I put in the needed effort, I am sure I can become a good writer in English.				
5	I can write essays that are relevant and appropriate to the assignment.				
6	I present my point of view or arguments accurately and effectively when writing in English.				
7	I am sure I can do well on writing courses even if they are difficult.				

**Table:** 

Items	Never	Rarely	Sometimes	Often	Always
1	I enjoy writing in English.				
2	Writing in English is very important to me.				
3	I always look forward to my ESL writing classes.				
4	I would like to spend lots of time learning to write in English.				
5	I would like to concentrate on learning to write in English more than any other topic.				
6	I actively think about what I have learned in my English writing class.				
7	I really try to learn how to write in English.				

## Footnotes

^1^ http://dialangweb.lancaster.ac.uk

## Appendices

Appendix A. L2 writing self-efficacy scale.

Appendix B. Writing Motivation Scale.
